# Supplementary material for: New evidence confirming the CD genomic constitutions of the tetraploid Avena species in the section Pachycarpa Baum
Source: PLoS One. 2021 Jan 8;16(1):e0240703. doi: 10.1371/journal.pone.0240703 (PMC7793304; doi:10.1371/journal.pone.0240703)
Supplement: S1 Raw images — (PDF) [file pone.0240703.s001.pdf]

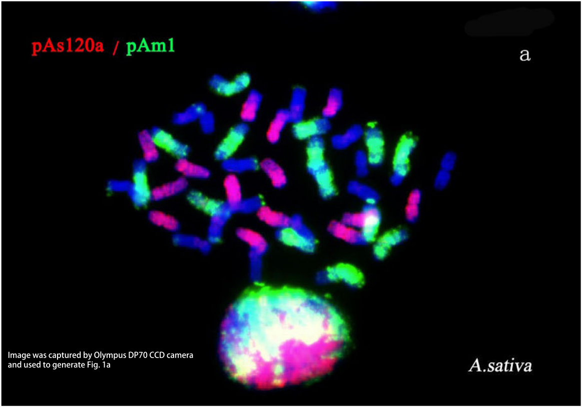

Image was captured by Olympus DP70 CCD camera
and used to generate Fig. 1a

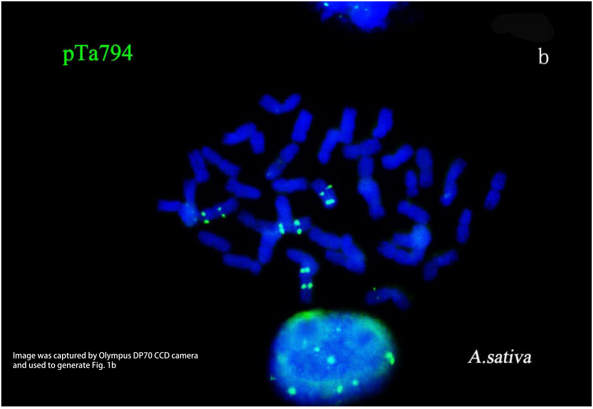

Image was captured by Olympus DP70 CCD camera
and used to generate Fig. 1b

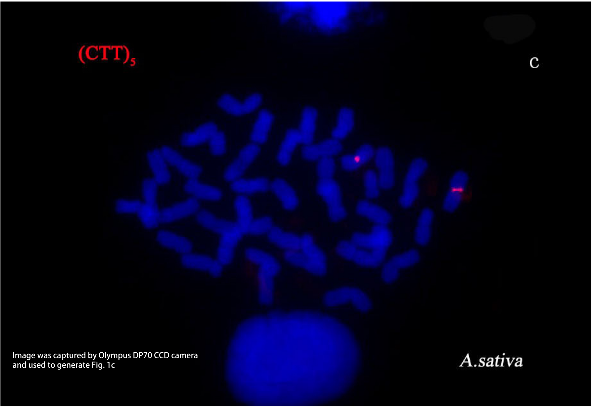

Image was captured by Olympus DP70 CCD camera and used to generate Fig. 1c

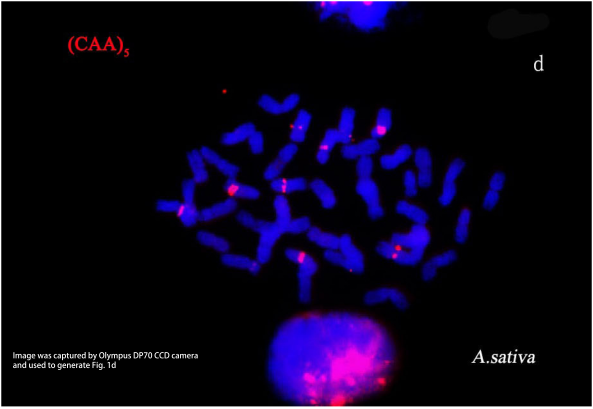

Image was captured by Olympus DP70 CCD camera
and used to generate Fig. 1d

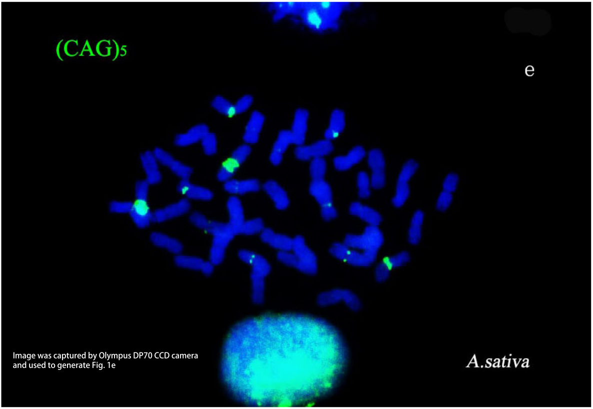

Image was captured by Olympus DP70 CCD camera and used to generate Fig. 1e

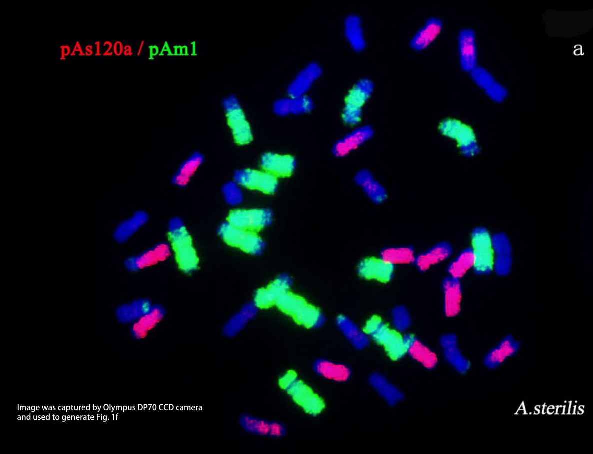
pAs120a / pAm1
a
Image was captured by Olympus DP70 CCD camera
and used to generate Fig. 1f
A.sterilis

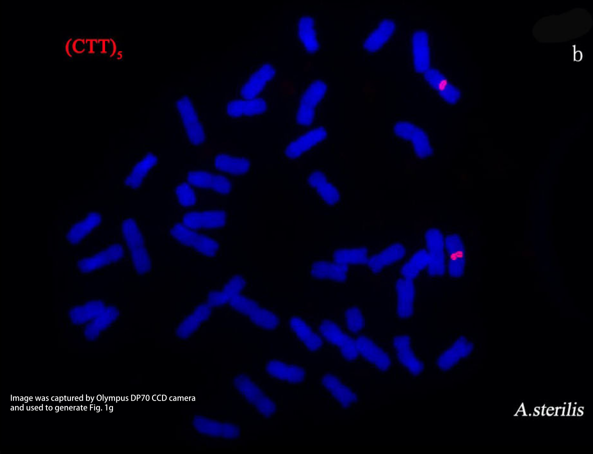

$(CTT)_5$
b
Image was captured by Olympus DP70 CCD camera
and used to generate Fig. 1g
A.sterilis

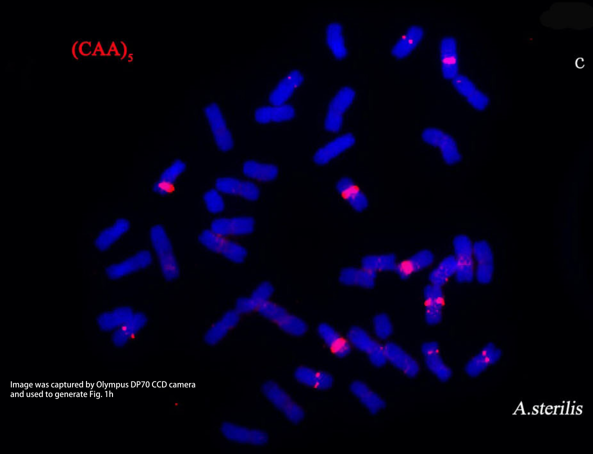
(CAA)5
c
A.sterilis
Image was captured by Olympus DP70 CCD camera
and used to generate Fig. 1h

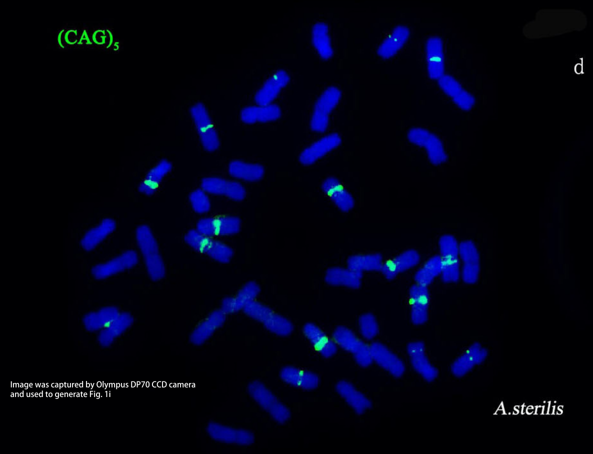

Image was captured by Olympus DP70 CCD camera
and used to generate Fig. 1i

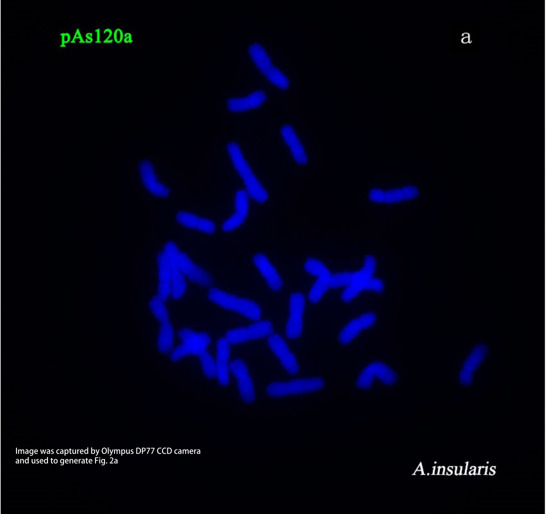

Image was captured by Olympus DP77 CCD camera
and used to generate Fig. 2a

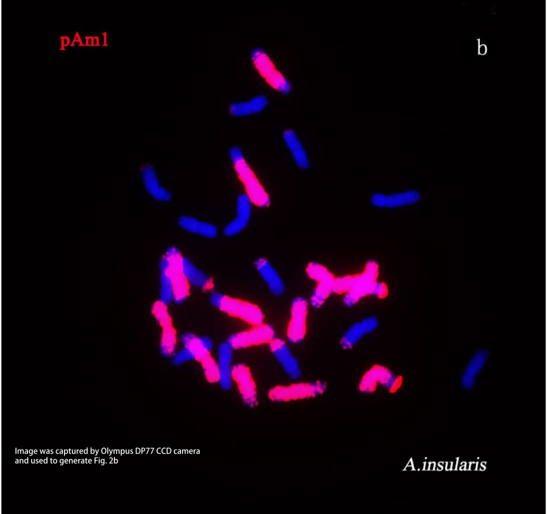

Image was captured by Olympus DP77 CCD camera and used to generate Fig. 2b

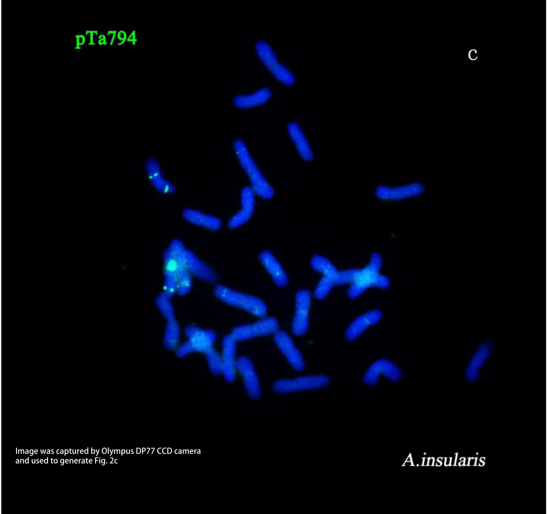

Image was captured by Olympus DP77 CCD camera
and used to generate Fig. 2c

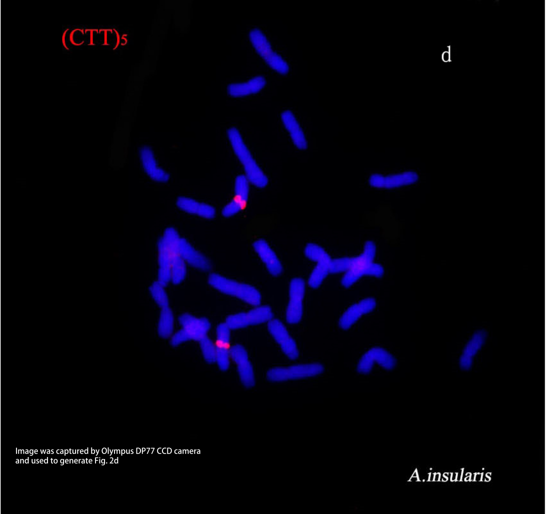

Image was captured by Olympus DP77 CCD camera
and used to generate Fig. 2d

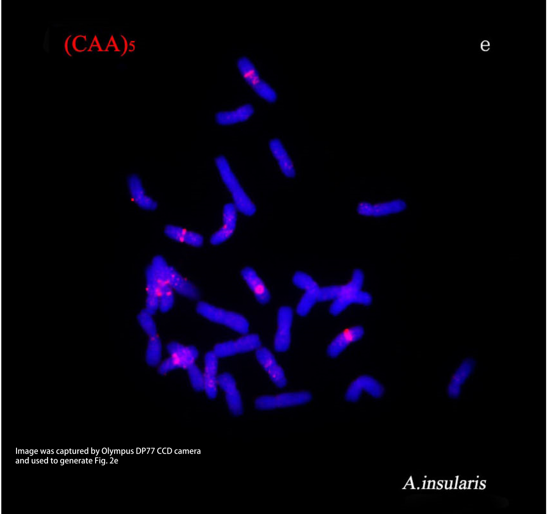

Image was captured by Olympus DP77 CCD camera
and used to generate Fig. 2e

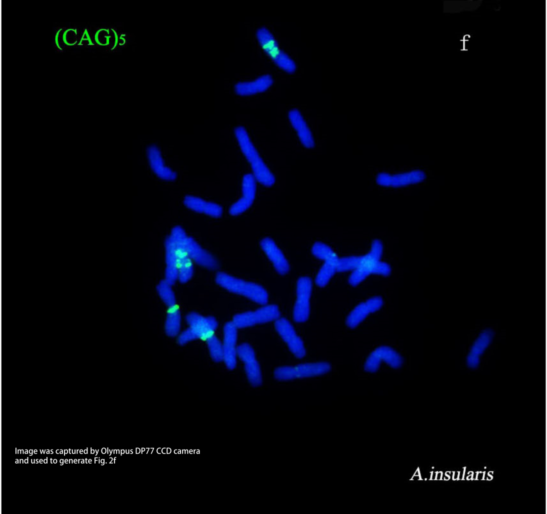

Image was captured by Olympus DP77 CCD camera
and used to generate Fig. 2f

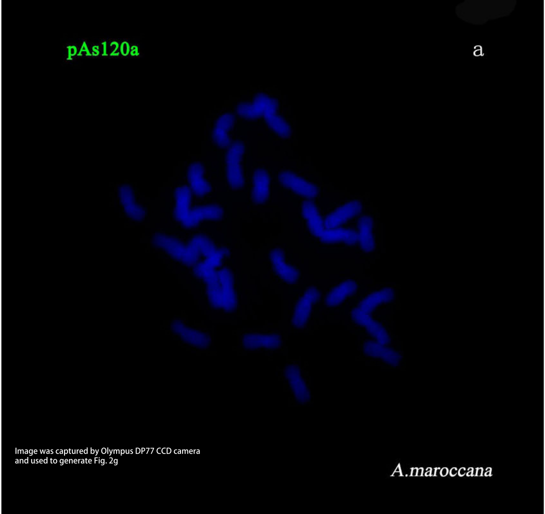
pAs120a
a
Image was captured by Olympus DP77 CCD camera and used to generate Fig. 2g
A.maroccana

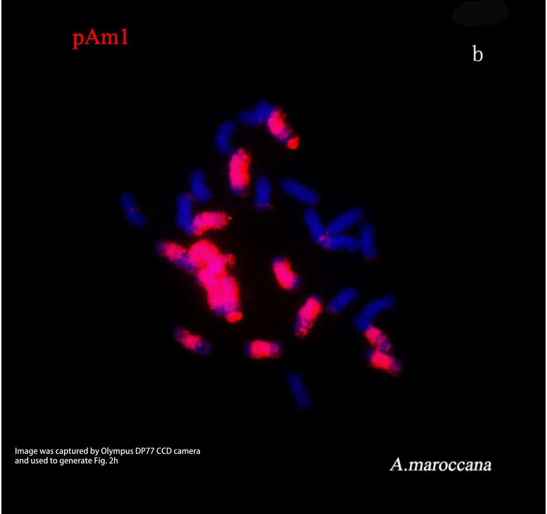

Image was captured by Olympus DP77 CCD camera
and used to generate Fig. 2h

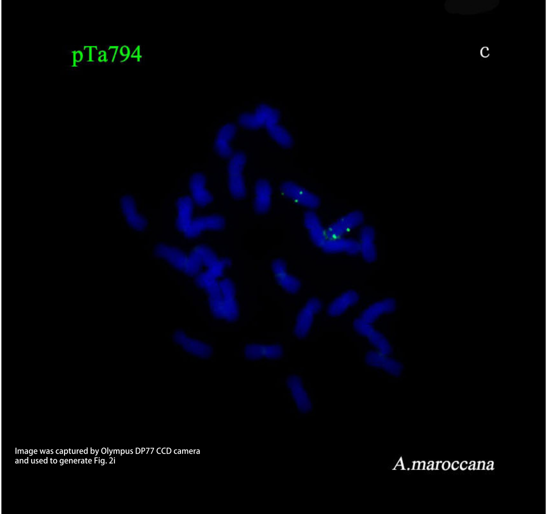

Image was captured by Olympus DP77 CCD camera and used to generate Fig. 2i

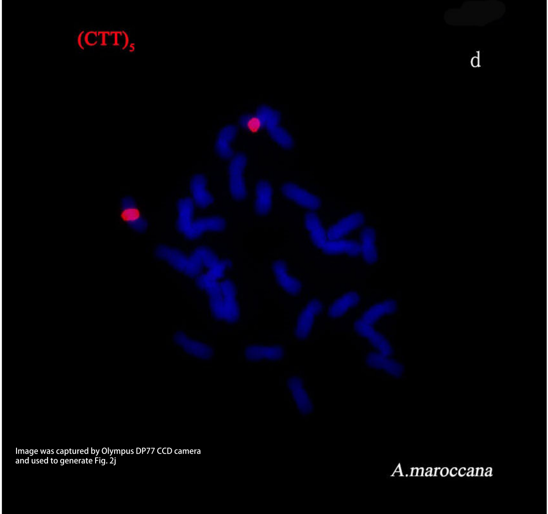

Image was captured by Olympus DP77 CCD camera and used to generate Fig. 2j

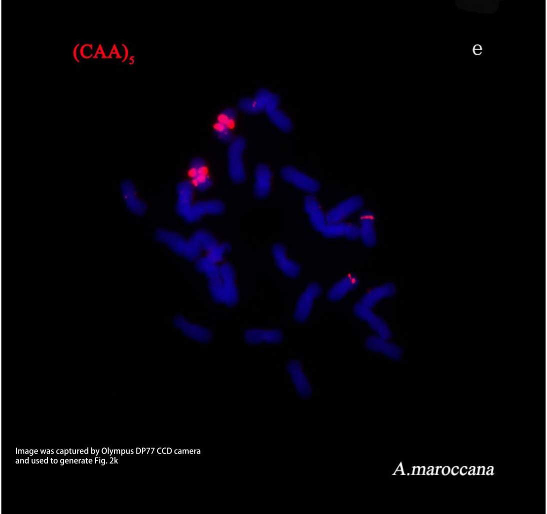

Image was captured by Olympus DP77 CCD camera and used to generate Fig. 2k

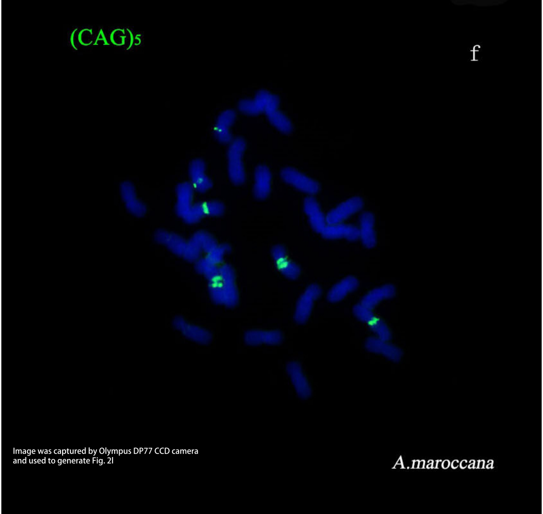

Image was captured by Olympus DP77 CCD camera and used to generate Fig. 2l

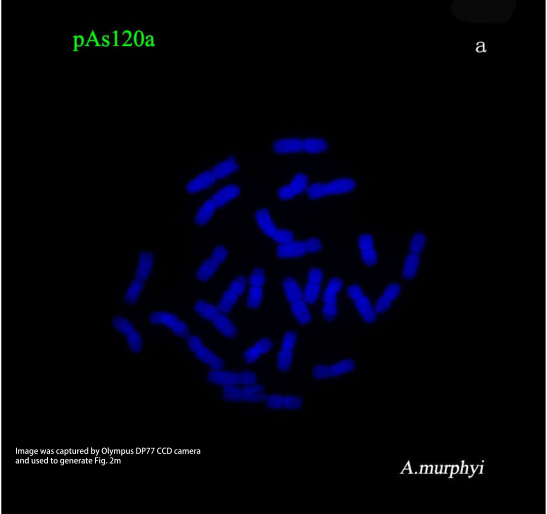

Image was captured by Olympus DP77 CCD camera
and used to generate Fig. 2m

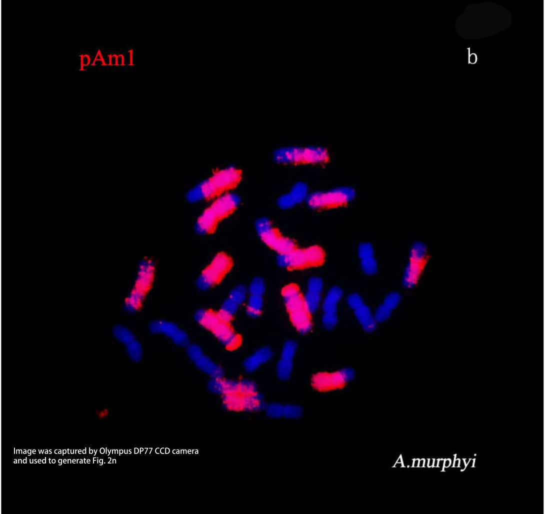

Image was captured by Olympus DP77 CCD camera
and used to generate Fig. 2n

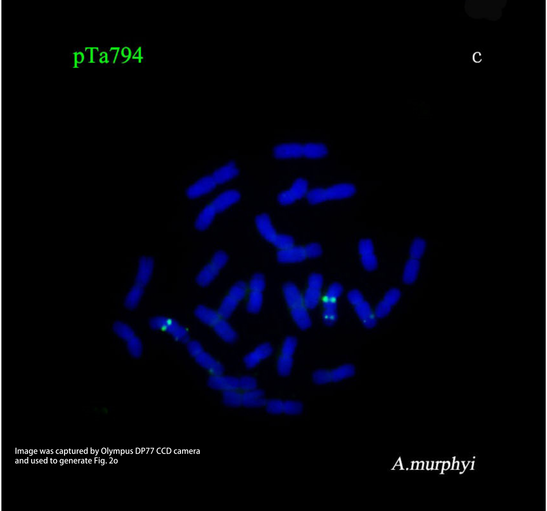

Image was captured by Olympus DP77 CCD camera and used to generate Fig. 2o

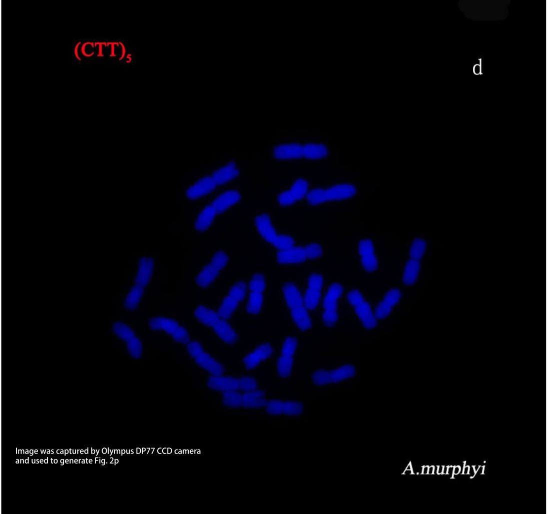

Image was captured by Olympus DP77 CCD camera
and used to generate Fig. 2p

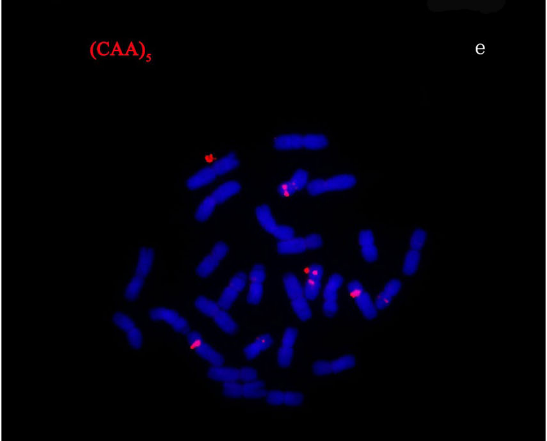

Image was captured by Olympus DP77 CCD camera
and used to generate Fig. 2q

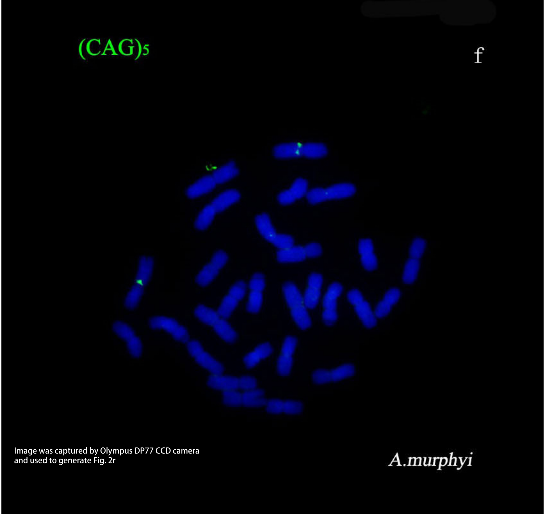

Image was captured by Olympus DP77 CCD camera
and used to generate Fig. 2r
